# Dermatomyositis with Eosinophils

**DOI:** 10.3390/dermatopathology10040039

**Published:** 2023-11-21

**Authors:** Isabella I. Sanchez, Henry O. Herrera, Ashley Elsensohn, Bonnie A. Lee, Christina N. Kraus

**Affiliations:** 1 School of Medicine, University of California Irvine, Irvine, CA 92697, USA; iisanche@hs.uci.edu; 2 School of Medicine, Case Western Reserve University, Cleveland, OH 44106, USA; hoh2@case.edu; 3 Departments of Dermatology and Pathology, Loma Linda University, Loma Linda, CA 92354, USA; 4 Department of Dermatology, University of California Irvine, Irvine, CA 92697, USA; bonniel6@hs.uci.edu (B.A.L.); ckraus@hs.uci.edu (C.N.K.)

**Keywords:** eosinophils, dermatomyositis, pruritus, connective tissue disease, diagnostic pitfall

## Abstract

Dermatomyositis is an idiopathic inflammatory myopathy that often presents with symmetric proximal skeletal muscle weakness and characteristic skin findings. Typical skin biopsy findings include vacuolar changes of the basal layer, increased dermal mucin, and a predominantly lymphocytic infiltrate. We report a case of dermatomyositis presenting as intensely pruritic papules and plaques, with initial histopathology being atypical of dermatomyositis due to the presence of eosinophils. The initial biopsy demonstrated a superficial dermatitis with eosinophils, initially thought to represent a drug eruption. A second biopsy of the same cutaneous manifestation was performed at a later time given high clinical suspicion for dermatomyositis and demonstrated a more classic vacuolar interface dermatitis with increased mucin and an absence of eosinophils. Notably, increased pruritus was specifically associated with the lesion that demonstrated tissue eosinophilia. The case illustrates the importance of considering tissue eosinophilia in the histologic presentation of dermatomyositis.

## 1. Introduction

Dermatomyositis is an autoimmune disorder affecting connective tissue, characterized by unique hallmark skin manifestations and weakness in proximal muscles. Since its initial documentation by Unverricht in 1887, dermatomyositis has been a subject of fascination for medical researchers and clinicians alike, spurring extensive research into its origin, epidemiology, and clinical presentation. Dermatomyositis has an estimated incidence of 9.63 per million persons and a prevalence of 21.42 per 100,000 persons [1]. It exhibits a diverse epidemiological landscape that encompasses individuals from various age groups, with a bimodal distribution for the age of diagnosis: juvenile dermatomyositis typically arises between 4–14 years, while in adults, it is seen predominantly between 40–60 years of age [2]. Furthermore, females are more frequently affected by dermatomyositis compared to males, with a 2:1 female-to-male ratio [2].

The pathogenesis of dermatomyositis involves an interplay between genetic, environmental, and autoimmune factors. Genotyping studies have demonstrated that major histocompatibility complex polymorphisms have been associated with the generation of autoantibodies, increasing the likelihood of developing dermatomyositis. For example, one study found that Human Leukocyte Antigen (HLA)-DRB1*04:01 and HLA-DRB1*12:02 are associated with an increased risk of producing anti-MDA5 antibodies [3]. Other autoantibodies are associated with dermatomyositis, including anti-Mi2, anti-NXP2, anti-TIF1, and SAE antibodies [2,4].

Additionally, environmental exposures play a role in dermatomyositis by triggering chronic immune activation in patients who are genetically susceptible. One study demonstrated that regions in the United States with higher UV indexes, such as the southwest, had a greater percentage of women with dermatomyositis. Moreover, these patients also demonstrated a greater percentage of anti-Mi-2 autoantibodies compared to regions with less UV exposure [5].

These findings correlate with another study that observed increased Mi-2 autoantigen protein levels in keratinocyte cell cultures that were exposed to UV radiation via protein translational effects [6]. Other factors such as smoking, viral or bacterial infections, and medications have been associated with triggering dermatomyositis as well [6,7]. Although dermatomyositis has an autoimmune component to its pathogenicity, the role of the autoantibodies in the disease is not fully understood.

The early activation of the complement cascade has been implicated in the pathogenesis of dermatomyositis. Studies have demonstrated that the membrane attack complex deposits on endomysial capillaries, which results in their imminent destruction and leads to ischemia, hypoperfusion, and microinfarction [2,8]. Additionally, interferons have been found to play a role in dermatomyositis by leading to the activation of the T cells and B cells that may be responsible for generating autoantibodies [2]. T-cells are also believed to be involved in the muscle inflammation found in dermatomyositis due to their presence in patient muscle biopsies [2,4].

Categorized as an idiopathic inflammatory myopathy closely linked to polymyositis, dermatomyositis frequently gives rise to a range of systemic complications. The diversity in its clinical presentation introduces complexities in its prompt identification and effective management. Dermatomyositis falls under the umbrella of idiopathic inflammatory myopathies, a diverse collection of autoimmune conditions. These myopathies are marked by chronic inflammation and an advancing decline in skeletal muscle strength. Dermatomyositis, while sharing similarities with other idiopathic inflammatory myopathies, possesses distinct clinical and pathological attributes that set it apart within this complex spectrum of diseases. Dermatomyositis is often accompanied by systemic complications such as interstitial lung disease, dysphagia, and proximal and symmetrical muscle weakness, making it a multisystem disorder with diverse clinical manifestations.

The cutaneous manifestations of dermatomyositis range from distinctively pathognomonic to nonspecific indicators. The presence of skin lesions serves as an instrumental tool in the diagnosis of dermatomyositis. The prototypical dermatological signs for dermatomyositis encompass Gottron’s sign, Gottron’s papules, and the heliotrope rash. Gottron’s papules are raised papular, sometimes scaly, infiltrated purplish lesions that can be found on the metacarpophalangeal joints, proximal interphalangeal joints, or distal interphalangeal joints [2,9].

Gottron’s sign consists of erythematous macules or patches that lay over extensor surfaces such as the elbows, knees, knuckles, and medial malleoli [2,9]. Another distinctive skin finding is the heliotrope rash, which consists of periorbital erythema with edema. This finding is most commonly located on the upper eyelids but can also appear on the cheeks and nose [2,9]. The shawl sign is characterized by symmetrical erythematous macules that extend from the nape of the neck down the posterior shoulders, reaching the upper back. Occasionally, these violaceous poikilodermatous patches can spread down to the lateral arm [2,9,10]. The holster sign is marked by a distinct, violaceous discoloration and is predominantly found on the outer areas of the hips and upper thighs and extends below the greater trochanter. Its symmetric presence on both sides mirrors the position of a leather holster, leading to its unique name, “Holster sign”. Its patterns, which can be meshed, livedoid, or linear, are considered highly specific for dermatomyositis [2,9]. Other cutaneous findings include atrophic dermal papules, poikiloderma, calcinosis cutis, and photosensitivity.

Histologically, dermatomyositis presents with distinctive features in both the skin and muscle tissues. However, some of its histological characteristics are shared by other connective tissue disorders. As such, correlation with clinical and laboratory findings is crucial for the accurate diagnosis of dermatomyositis. The specific lesions of dermatomyositis, such as Gottron’s papules, Gottron’s sign, and the heliotrope rash, are predominantly characterized by interface dermatitis when observed histologically [11].

Additional findings consist of basal layer vacuolar alterations coupled with hyperkeratosis, telangiectasia, pigment incontinence, upper dermal edema, epidermal atrophy, thickening of the basement membrane, and mucin deposition in the papillary dermis [11,12]. Another study found the presence of multiple venule emboli with sparse inflammatory infiltration, vasculopathy, and epidermal necrosis in skin biopsies of ulcerative Gottron’s papules and Gottron’s sign [13] CD4+ T lymphocytic infiltrates and melanophages may be present on the superficial dermis, as well as neutrophils in the papillary dermis [11,12,13]. Occasionally, eosinophils can be seen within the mucinous stroma [11,12]. The presence of CD123+ plasmacytoid dendritic cells have also been found in clusters in the superficial dermis and in the epidermis [4,13].

Eosinophil infiltration is a key histopathological finding used to aid in the diagnosis of eosinophilic dermatoses. Conversely, the absence or scarcity of tissue eosinophilia has been traditionally thought to be characteristic of several inflammatory skin diseases, such that its presence may lead to ruling out the diagnosis. Recent studies have captured the presence of eosinophils in traditionally eosinophil-poor skin diseases, such as dermatomyositis. The release of eosinophil granule proteins, such as major basic protein, eosinophil cationic protein, and eosinophil-derived neurotoxin, stimulate nerve cells that lead to the sensation of pruritus and mediate damage to tissues [14]. Other mediators, such as IL-4, IL-13, and IL-31, have also been shown to cause itch in various dermatologic conditions [15]. Although not commonly reported, the presence of eosinophils in dermatomyositis has been previously reported in scalp, arm, and hand biopsies [12,16]. Notably, eosinophils were observed in 44% of dermatomyositis skin biopsies in a study by Khanna et al. [16]. Herein, we report a case of dermatomyositis presenting initially with dermal eosinophils and later biopsy showing a more classic vacuolar interface dermatitis.

## 2. Case Report

**Figure 1:**
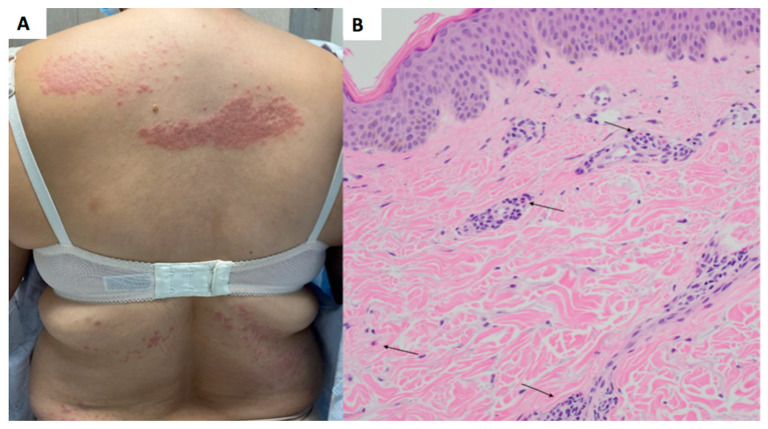
Upper back papules and plaques showing superficial perivascular dermatitis and eosinophils. (**A**) Clinical presentation, back. (**B**) Hematoxylin and eosin (H&E) stain, 20× magnification. Site: back. There is a superficial perivascular mixed infiltrate with eosinophils (highlighted by arrows) and increased dermal mucin.

**Figure 2:**
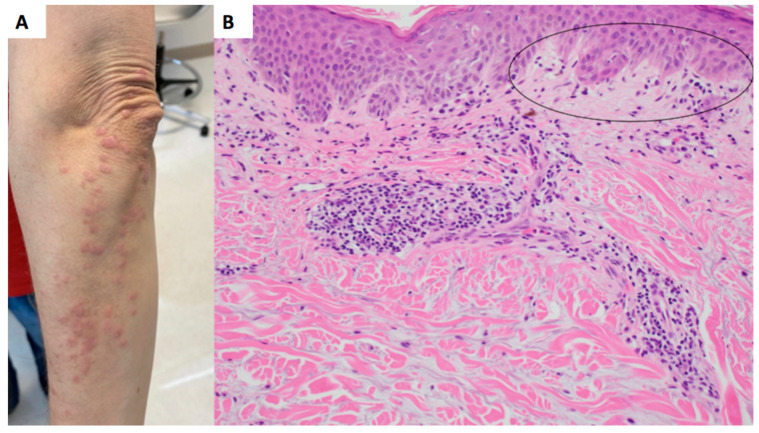
Left arm papules and plaques with interface dermatitis, increased mucin, and an absence of eosinophils. (**A**) Clinical presentation, arm. (**B**) Hematoxylin and eosin (H&E) stain, 20× magnification. Site: arm. There is a subtle vacuolar interface (circled) with focal pigment dropout, a superficial perivascular lymphohistiocytic infiltrate and increased dermal mucin.

A 53-year-old female with an outside diagnosis of dermatomyositis with anti-NXP-2 positivity presented with pruritic and progressive papules and plaques on the upper and lower back (Figure 1A) and bilateral upper extremities (Figure 2A). Prior to presentation, an outside autoimmune work-up had been performed that was positive for ANA 1:160 in a speckled pattern and anti-NXP-2. ESR, CRP, Aldolase, and antibodies for beta 2 gylycoprotein, lupus anticoagulant, RNP, Smith, SmRNP, SSA, SSB, Scl70, Jo1, Centromere B, Chromatin, Ribosomal P, and anti-cardiolipin were negative. Anti-double-stranded DNA was equivocal. Two prior outside skin biopsies showed a vacuolar-interface dermatitis. Malignancy work-up was negative. Given the patient’ above serologies in the context of a clinical rash and muscle weakness, the patient was diagnosed with dermatomyositis.

At the time of presentation to our clinic, a biopsy of the upper back was performed, which showed a superficial perivascular dermatitis with eosinophils (Figure 1B). Histologically, it was favored to represent a drug eruption, although clinically, the patient had not been placed on any new medications. A CBC was unremarkable for peripheral eosinophilia. She was treated with fluocinonide 0.1% ointment twice daily as needed for the rash, which mildly improved. A subsequent biopsy of the left arm was performed approximately 3 months later, showing a subtle interface dermatitis with increased dermal mucin (Figure 2B) and an absence of eosinophils. Retrospectively, a mucin stain was performed of the upper back biopsy, demonstrating an increase in mucin the upper dermis.

The histologic and clinical findings at this time were determined to be consistent with dermatomyositis, and the patient was started on Intravenous Immunoglobulin (IVIG) 2 g/kg per monthly with improvement in rash.

## 3. Discussion

This report adds an additional case to support the finding that tissue eosinophilia in dermatomyositis may be more common than previously thought. It also highlights the potential association of tissue eosinophils and pruritus in dermatomyositis. Pruritus has been identified as a significant symptom in patients with dermatomyositis, appearing in more than 90% of patients in one study [17]. The distribution of pruritus in dermatomyositis usually follows the typical distribution of the skin lesions afflicting the trunk, arms, face, and scalp [18]. Of interest, our patient reported more itch associated with the lesions on her back where the biopsy demonstrated prominent eosinophils. This is consistent with previous findings that reported increased pruritus in sites that contained eosinophils in the inflammatory infiltrate [15,16]. A more classic connective tissue histology without eosinophils was observed on the upper extremities of our patient. In one study, the most common biopsy sites were on the hands and arms, but the anatomic location and eosinophil count were not correlated [16]. It is uncertain at this time whether anatomic location makes a difference in eosinophil density in dermatomyositis.

The presence of eosinophils in dermatomyositis has been previously reported in scalp, arm, and hand biopsies [12,16]. Notably, eosinophils were observed in 44% of dermatomyositis skin biopsies in a study by Khanna et al. [16]. IL-31 especially has been found to play a role in the pruritus associated with dermatomyositis [15]. A study by Kim et al. [17] demonstrated that skin from lesional dermatomyositis had increased gene expression of IL-31 compared to dermatomyositis patients without skin lesions and control patients. Additionally, IL-31 mRNA expression was positively correlated with a visual analog scale itch score [17]. Notably, IL-31 may also play a role in the recruitment of eosinophils [19,20], with in vitro studies demonstrating that IL-31 significantly upregulated the expression of CD18 on eosinophils. CD18 plays a role in eosinophil adhesion, rolling, and transmigration, allowing eosinophils to migrate to the source of inflammation [21]. The presence of eosinophils in dermatomyositis may be influenced by the elevated levels of IL-31 seen in the condition. However, a definitive correlation necessitates further comprehensive research.

The presence of eosinophils in dermatomyositis is important to highlight since it can present a diagnostic pitfall. This was demonstrated in our case, with the initial conclusion favored was that of a drug eruption rather than dermatomyositis. Only because of high clinical suspicion for dermatomyositis was a subsequent biopsy performed, which only several months later showed the more classic histologic features of connective tissue disease. This study highlights that tissue eosinophilia itself should not be used to rule out a diagnosis if there are other supportive histologic findings or if there is a high clinical suspicion for the diagnosis in question.

Other classically eosinophil-poor dermatological conditions can present with eosinophils in biopsy. A study on lupus panniculitis and morphea profunda demonstrated the presence of eosinophils in skin biopsies. In lupus panniculitis, 8 out of 33 (24%) patients had eosinophils in their biopsy, with one specimen having greater than 10 eosinophils per HPF. The patient did not have a known clinical cause of eosinophilia and had a normal peripheral eosinophil value [22]. In morphea profunda, eosinophils were observed in 13 out of 53 patients (25%). Eosinophils were not detected in the lymphoid aggregates in biopsies from both conditions [22]. These findings diverge from the typical histological findings of lupus panniculitis and morphea profunda, which primarily consist of lymphoplasmacytic infiltrates [23].

Another dermatologic condition, hypertrophic lichen planus, has been shown to have a wide histopathologic spectrum. In one study, biopsies ranged from having little to no eosinophils, while others had a larger number of eosinophils present [24]. Additionally, it was found that 13 out of 63 (20.6%) biopsies from patients with hypertrophic lichen planus had greater than 10 eosinophils per 10–20 fields. The presence of eosinophils in these biopsies made them histopathologically indiscernible from a lichenoid drug eruption [24].

Several studies have also examined the presence of eosinophils in psoriasis. Psoriasis vulgaris is typically diagnosed based on characteristic histological findings such as epidermal hyperplasia, hyperkeratosis, confluent parakeratosis, an absent or reduced granular layer, and an elongation of epidermal rete ridges. Inflammatory infiltrates consisting of neutrophils in the stratum corneum and epidermis, mononuclear infiltrates in the epidermis, and leukocyte infiltration into the dermis can also be found [25,26]. Eosinophils tend to be an uncommon histological finding in psoriasis. However, studies have demonstrated their presence in psoriasis patients’ skin biopsies. A study by Lundin et al. found the presence of activated eosinophils in 11 of 15 psoriasis biopsies [27]. An additional study by Rosa et al. found dermal eosinophils in 15 out of 85 (18%) psoriasis patients’ biopsies, with a maximum of 3 eosinophils in the biopsy sections [28]. The low number of eosinophils, specifically less than three per biopsy, led the authors to conclude that eosinophils are a rare histological finding in psoriasis and argue against the diagnosis [28]. However, a study by Chau et al. found that 25 out of 51 (49%) of their psoriasis biopsy specimens contained dermal eosinophils [29]. Building on these findings, another study by Penn et al. found that 23 out of 50 (46%) psoriasis vulgaris biopsies contained eosinophils, which were comparable with the findings from Chau et al. [25,29]. Additionally, studies have demonstrated that IL-31 appears to contribute to the induction of pruritus in psoriasis. However, IL-31’s relationship with pruritus intensity is not well established [30]. A study by Purzycka-Bohdan et al. found substantially elevated levels of IL-31 in psoriasis patients compared to healthy controls, with 97.4% of psoriasis demonstrating pruritus. However, there was no correlation between IL-31 serum levels, pruritus intensity, and psoriasis severity [31].

Several factors may contribute to the variability of tissue eosinophilia, such as chronicity and anatomic site. Dermatomyositis, while characterized by eosinophils and itch in its early stages, may demonstrate a decrease in eosinophils over time. Similar observations have been suggested in morphea, which has more eosinophils in earlier lesions [32]. Anatomic sites may also affect tissue eosinophilia, with prior studies showing more eosinophils in scalp dermatomyositis [16]. Additional studies are needed to observe the time course in dermatomyositis when tissue eosinophilia is present, as well as the clinical-pathologic correlation of eosinophils and pruritus.

## 4. Conclusions

In summary, the presence of eosinophils on skin biopsy in a patient with suspected dermatomyositis should not militate against the diagnosis of dermatomyositis. Given that eosinophilic dermatoses have been associated with pruritus via the release of factors such as granule proteins and cytokines, one might consider the possibility of an association between pruritus- and eosinophil-positive dermatomyositis. This could have treatment implications as the pathway of itch in dermatomyositis is further investigated.

## Data Availability

Data available on request due to restrictions eg privacy or ethical. The data presented in this study are available on request from the corresponding author. The data are not publicly available due to patient privacy.

## References

[1] Bendewald M.J., Wetter D.A., Li X., Davis M.D. (2010). Incidence of dermatomyositis and clinically amyopathic dermatomyositis: A population-based study in Olmsted County, Minnesota. Arch. Dermatol..

[2] DeWane M.E., Waldman R., Lu J. (2020). Dermatomyositis: Clinical features and pathogenesis. J. Am. Acad. Dermatol..

[3] Chen Z., Wang Y., Kuwana M., Xu X., Hu W., Feng X., Wang H., Kimura A., Sun L. (2017). HLA-DRB1 Alleles as Genetic Risk Factors for the Development of Anti-MDA5 Antibodies in Patients with Dermatomyositis. J. Rheumatol..

[4] Lundberg I.E., Fujimoto M., Vencovsky J., Aggarwal R., Holmqvist M., Christopher-Stine L., Mammen A.L., Miller F.W. (2021). Idiopathic inflammatory myopathies. Nat. Rev. Dis. Prim..

[5] Love L.A., Weinberg C.R., McConnaughey D.R., Oddis C.V., Medsger T.A., Reveille J.D., Arnett F.C., Targoff I.N., Miller F.W. (2009). Ultraviolet radiation intensity predicts the relative distribution of dermatomyositis and anti-Mi-2 autoantibodies in women. Arthritis Rheum..

[6] Burd C.J., Kinyamu H.K., Miller F.W., Archer T.K. (2008). UV radiation regulates Mi-2 through protein translation and stability. J. Biol. Chem..

[7] Bax C.E., Maddukuri S., Ravishankar A., Pappas-Taffer L., Werth V.P. (2021). Environmental triggers of dermatomyositis: A narrative review. Ann. Transl. Med..

[8] Dalakas M.C. (2010). Immunotherapy of myositis: Issues, concerns and future prospects. Nat. Rev. Rheumatol..

[9] Muro Y., Sugiura K., Akiyama M. (2015). Cutaneous Manifestations in Dermatomyositis: Key Clinical and Serological Features—A Comprehensive Review. Clin. Rev. Allergy Immunol..

[10] Bogdanov I., Kazandjieva J., Darlenski R., Tsankov N. (2018). Dermatomyositis: Current concepts. Clin. Dermatol..

[11] Aussy A., Boyer O., Cordel N. (2017). Dermatomyositis and Immune-Mediated Necrotizing Myopathies: A Window on Autoimmunity and Cancer. Front. Immunol..

[12] Jasso-Olivares J., Diaz-Gonzalez J.M., Miteva M. (2018). Horizontal and vertical sections of scalp biopsy specimens from dermatomyositis patients with scalp involvement. J. Am. Acad. Dermatol..

[13] Cao H., Xia Q., Pan M., Zhao X., Li X., Shi R., Zhou M., Ding X., Kuwana M., Zheng J. (2016). Gottron Papules and Gottron Sign with Ulceration: A Distinctive Cutaneous Feature in a Subset of Patients with Classic Dermatomyositis and Clinically Amyopathic Dermatomyositis. J. Rheumatol..

[14] Long H., Zhang G., Wang L., Lu Q. (2016). Eosinophilic Skin Diseases: A Comprehensive Review. Clin. Rev. Allergy Immunol..

[15] Radonjic-Hoesli S., Brüggen M.C., Feldmeyer L., Simon H.U., Simon D. (2021). Eosinophils in skin diseases. Semin. Immunopathol..

[16] Khanna U., Vaughan H., North J., Haemel A. (2021). Quantitative Assessment of Eosinophils in Dermatomyositis Skin Biopsies with Correlation of Eosinophils to Pruritus and Other Clinical Features. Am. J. Dermatopathol..

[17] Kim H.J., Zeidi M., Bonciani D., Pena S.M., Tiao J., Sahu S., Werth V.P. (2018). Itch in dermatomyositis: The role of increased skin interleukin-31. Br. J. Dermatol..

[18] Kim H.J. (2021). Pruritus in autoimmune connective tissue diseases. Ann. Transl. Med..

[19] Gibbs B.F., Patsinakidis N., Raap U. (2019). Role of the Pruritic Cytokine IL-31 in Autoimmune Skin Diseases. Front. Immunol..

[20] Kunsleben N., Rüdrich U., Gehring M., Novak N., Kapp A., Raap U. (2015). IL-31 Induces Chemotaxis, Calcium Mobilization, Release of Reactive Oxygen Species, and CCL26 in Eosinophils, Which Are Capable to Release IL-31. J. Investig. Dermatol..

[21] Cheung P.F., Wong C.K., Ho A.W., Hu S., Chen D.P., Lam C.W. (2010). Activation of human eosinophils and epidermal keratinocytes by Th2 cytokine IL-31: Implication for the immunopathogenesis of atopic dermatitis. Int. Immunol..

[22] Peters M.S., Su W.P. (1991). Eosinophils in lupus panniculitis and morphea profunda. J. Cutan. Pathol..

[23] Wick M.R. (2017). Panniculitis: A summary. Semin. Diagn. Pathol..

[24] Alomari A., McNiff J.M. (2014). The significance of eosinophils in hypertrophic lichen planus. J. Cutan. Pathol..

[25] Penn L., Brinster N.K. (2019). Eosinophils Among the Histological Features of Psoriasis. Am. J. Dermatopathol..

[26] Ayala-Fontánez N., Soler D.C., McCormick T.S. (2016). Current knowledge on psoriasis and autoimmune diseases. Psoriasis.

[27] Lundin A., Fredens K., Michaëlsson G., Venge P. (1990). The eosinophil granulocyte in psoriasis. Br. J. Dermatol..

[28] Rosa G., Fernandez A.P., Schneider S., Billings S.D. (2017). Eosinophils are rare in biopsy specimens of psoriasis vulgaris. J. Cutan. Pathol..

[29] Chau T., Parsi K.K., Ogawa T., Kiuru M., Konia T., Li C.S., Fung M.A. (2017). Psoriasis or not? Review of 51 clinically confirmed cases reveals an expanded histopathologic spectrum of psoriasis. J. Cutan. Pathol..

[30] Kaczmarska A., Kwiatkowska D., Skrzypek K.K., Kowalewski Z.T., Jaworecka K., Reich A. (2023). Pathomechanism of Pruritus in Psoriasis and Atopic Dermatitis: Novel Approaches, Similarities and Differences. Int. J. Mol. Sci..

[31] Purzycka-Bohdan D., Gleñ J., Zabłotna M., Nedoszytko B., Szczerkowska-Dobosz A., Sokołowska-Wojdyło M., Rêbała K., Nowicki R.J. (2021). Significance of interleukin-31 (IL-31) gene polymorphisms and IL-31 serum level in psoriasis in correlation with pruritus. Postepy Dermatol. Alergol..

[32] Kim J., Chung K.B., Lee Y.I., Kim J., Lee J.H. (2021). Clinical characteristics and histopathologic changes of morphea: A single-center, retrospective study of 137 patients. J. Am. Acad. Dermatol..

